# Toxic chemical element barium content in different biosubstrates of children with congenital heart diseases

**DOI:** 10.1186/1749-8090-8-S1-O140

**Published:** 2013-09-11

**Authors:** O Koval, I Mokryk, N Nagorna, G Dubova, O Dudchak, V Volodyn, N Usenko, O Muzychyn, A Nechiporchuk, A Novak

**Affiliations:** 1 Pediatric Subdepartment of Internship and Postgraduate Education Faculty of Donetsk National Medical University n.a. M. Gorkiy, Donetsk, Ukraine; 2 Department of Cardiosurgery, Cardiology and Rehabilitation for Children, Government Institution “Institute of Urgent and Recovery Surgery named after V. K. Gusak National Academy of Medical Science of Ukraine”, Donetsk, Ukraine

## Background

Number of studies indicates on possible role of toxic substances in development of heart malformations during cardiogenesis. The aim of our research is studying of toxic element barium content in different biosubstrates of children with CHD.

## Methods

29 patients (17 (58,6%) boys and 12 (41,6%) girls), aged from 14 days to 17 years (medium age: 26 month±2 month) old with CHD were examined by the spectral analysis of toxic element barium in intraoperative (23) and autopsy (6) biopsies of endocardium (22), myocardium (26), pericardium (7), aorta (10), skin (8), subcutaneous fat (8), intercostal muscles (24), fascia (2), kidneys (5), brain (2) and hair (9) by methods of the atomic emission spectrometry in the inductively coupled plasma and atomic absorption spectrometry with electrothermal atomization.

## Results

A comparative analysis of barium level in different biosubstrates demonstrated it’s highest content (числа) in muscle tissue (myocardium, smooth muscle of the vessel walls, cross-striated rib muscles). Whereas in other tissues the concentration was decreased or absent. Barium content exceeded toxic level in heart tissues of 72.4% of patients. The excess degree of barium content depended on biopsy topics, namely, in aorta coarctation place, valve atresia place, septal defect its level was in two, ten or more times higher than in normal heart area or great vessel area. Dependence between the barium excess degree content in the heart tissue and severity of malformation or its combinations was also established: children with exceeding level of barium in the heart tissues had significantly more (p <0.05) of combined complex CHD.

## Conclusions

Barium affinity to the myocardium, blood vessels smooth muscles and striated skeletal muscles was confirmed. Spectrum of barium content depends on the type and severity of the defect and biopsy point. Received data indicate on possible role of barium in cardiogenesis malformation, that needs deeper investigation.

